# Measuring directed functional connectivity using non-parametric directionality analysis: Validation and comparison with non-parametric Granger Causality

**DOI:** 10.1016/j.neuroimage.2020.116796

**Published:** 2020-09

**Authors:** Timothy O. West, David M. Halliday, Steven L. Bressler, Simon F. Farmer, Vladimir Litvak

**Affiliations:** a Nuffield Department of Clinical Neurosciences, John Radcliffe Hospital, University of Oxford, Oxford OX3 9DU, UK; b Centre for Mathematics and Physics in the Life Sciences and Experimental Biology, Department of Computer Science, Gower Street, London, WC1E 6BT, UK; c Wellcome Centre for Human Neuroimaging, UCL Queen Square Institute of Neurology, London, WC1N 3AR, UK; d Department of Electronic Engineering and York Biomedical Research Institute, University of York, YO10 5DD, UK; e Centre for Complex Systems and Brain Sciences, Florida Atlantic University, 777 Glades Road, Florida, USA; f Department of Neurology, National Hospital for Neurology & Neurosurgery, Queen Square, London, WC1N 3BG, UK; g Department of Clinical and Movement Neurosciences, Institute of Neurology, UCL, London, WC1N 3BG, UK

**Keywords:** Functional connectivity, Directionality, EEG, MEG, Signal-to-noise, Volume conduction, Neural networks, Computational neuroscience, Multimodal data, Local field potentials

## Abstract

**Background:**

‘Non-parametric directionality’ (NPD) is a novel method for estimation of directed functional connectivity (dFC) in neural data. The method has previously been verified in its ability to recover causal interactions in simulated spiking networks in Halliday et al. (2015).

**Methods:**

This work presents a validation of NPD in continuous neural recordings (e.g. local field potentials). Specifically, we use autoregressive models to simulate time delayed correlations between neural signals. We then test for the accurate recovery of networks in the face of several confounds typically encountered in empirical data. We examine the effects of NPD under varying: a) signal-to-noise ratios, b) asymmetries in signal strength, c) instantaneous mixing, d) common drive, e) data length, and f) parallel/convergent signal routing. We also apply NPD to data from a patient who underwent simultaneous magnetoencephalography and deep brain recording.

**Results:**

We demonstrate that NPD can accurately recover directed functional connectivity from simulations with known patterns of connectivity. The performance of the NPD measure is compared with non-parametric estimators of Granger causality (NPG), a well-established methodology for model-free estimation of dFC. A series of simulations investigating synthetically imposed confounds demonstrate that NPD provides estimates of connectivity that are equivalent to NPG, albeit with an increased sensitivity to data length. However, we provide evidence that: i) NPD is less sensitive than NPG to degradation by noise; ii) NPD is more robust to the generation of false positive identification of connectivity resulting from SNR asymmetries; iii) NPD is more robust to corruption via moderate amounts of instantaneous signal mixing.

**Conclusions:**

The results in this paper highlight that to be practically applied to neural data, connectivity metrics should not only be accurate in their recovery of causal networks but also resistant to the confounding effects often encountered in experimental recordings of multimodal data. Taken together, these findings position NPD at the state-of-the-art with respect to the estimation of directed functional connectivity in neuroimaging.

## Highlights

• Non-parametric directionality (NPD) is a novel directed connectivity measure.
• NPD estimates are equivalent to estimates of Granger causality but are more robust to signal confounds.
• Multivariate extensions of NPD can correctly identify signal routing.

## Abbreviations

dFC: Directed functional connectivity
EEG: Electroencephalogram
LFP: Local field potential
MEG: Magnetoencephalogram
MVAR: Multivariate autoregressive (model)
NPD: Non-parametric directionality
(mv) NPG: (multivariate) Non-parametric estimator of Granger causality
SMA: Supplementary motor area
SNR: Signal-to-noise ratio
STN: Subthalamic Nucleus

## 1 Introduction

Questions regarding the causal relationships between anatomically connected regions of the brain have become of fundamental importance across many domains of neuroscience (Sporns, 2010; Swanson, 2012). A novel method for estimating directed functional connectivity (dFC), termed non-parametric directionality (NPD), has been recently described in Halliday (2015). This method has been demonstrated to yield physiological insights into the connectivity of the cortico-basal-ganglia network when applied to (continuous) field recordings made in rodents (West et al., 2018). In this work we evaluate NPD’s performance at recovering known patterns of connectivity in the face of several confounding factors and compare it with another popularly used measure – the estimation of Granger causality.

Functional connectivity is based on a description of the statistical dependencies between different neural signals and is typically estimated through time or frequency domain correlations (Bastos and Schoffelen, 2016; Friston, 2011). Magnitude squared coherence, equivalent to a frequency domain coefficient of correlation, has been widely adopted as the estimator of choice for functional connectivity in the neuroimaging community (Brillinger, 1975; Halliday et al., 1995). Undirected measures of functional connectivity (such as coherence) are symmetrical, giving no indication of the temporal precedence of correlations, a property understood to be a necessary result of causation in time evolving systems (Wiener, 1956), nor the predictability of one time series from that of the other. dFC aims to estimate statistical asymmetries in the correlated activity of a set of signals in order to infer the causal influence (or predictability) of one signal over another. Similar to the role played by coherence in measuring undirected functional connectivity, Wiener-Granger causality has emerged as a first-choice estimator of directed connectivity due to its well established theoretical basis (Bressler and Seth, 2011; Ding et al., 2006) and its successful application to questions concerning causal networks inferred from large-scale neural recordings (e.g. Brovelli et al., 2004; Richter et al., 2018).

Estimates of dFC are most frequently computed in the literature using methods estimating Granger causality (Dhamala et al., 2008a; Geweke, 1982; Granger, 1969; Kamiński et al., 2001). Granger causality is expressed in terms of the capacity of the information in the past of one signal, *X*, to predict the future of another signal, *Y*. Granger (1969) introduced a straightforward method of estimation through the comparison of an autoregressive model by which the explained variance of *Y* is compared between that of a ‘full’ model (i.e. accounting for the past of *X* and *Y*) with that of a restricted model (i.e. *Y* only). If a prediction of the future of *Y* is aided by information from the past of *X*, then *X* is said to ‘Granger-cause’ *Y*. The method requires factoring out the autoregressive component of the signal (i.e. the ‘restricted’ model) to avoid trivial correlations that occur simply due to the periodicity in the signals.

Efforts to estimate Granger causality without resorting to autoregressive models have resulted in an extension of the method termed non-parametric Granger causality (NPG), which avoids the estimation of transfer functions from multivariate autoregressive (MVAR) coefficients (Dhamala et al., 2008a). In NPG, transfer functions and noise covariances are estimated through the spectral factorization of (non-parametrically derived) Fourier coefficients rather from MVAR model parameters. Here, we directly compare NPG with NPD as an estimator of dFC. Both methods share the property of being non-parametric (model-free) approaches which can be derived from identical spectral transforms made either via Fourier or wavelet techniques.

NPD is founded on the same principles of causality as Granger, namely that temporally lagged *dependencies* indicate causal direction. NPD works by decomposing the coherence into three temporally independent components separated by the relative lag of the dependencies between the signals: 1) forward lagged; 2) reverse lagged; and 3) instantaneously correlated. Rather than using a naïve cross-correlation estimator that is susceptible to spurious peaks resulting from the individual signals’ autocorrelations, NPD takes an approach akin to the factoring out of a ‘restricted’ model (i.e. of *Y* only) used in Granger. This is achieved through a process of spectral pre-whitening which acts to bring the individual signal’s spectra closer to white-noise but preserves the correlations between them. In the original paper (Halliday, 2015), the method was validated using a simple three node network with each node’s dynamics simulated using a conductance model of a spiking neurone in order to generate a series of discrete point processes. The authors demonstrated that NPD was successful in recovering the connectivity from a range of simulated architectures. Furthermore, the method was applied to spike timings (a point process) recorded from muscle spindle and shown to yield physiologically plausible estimations of causality. Our recent work has extended the application of NPD to continuous local field potential (LFP) recordings made from an *in vivo* preparation of the cortico-basal ganglia system (West et al., 2018).

Estimation of empirical dFC in continuous neural recordings such as the LFP or magneto/electroencephalogram (M/EEG) is complicated by a number of factors. These include: low and possibly unequal signal-to-noise ratios (SNRs), instantaneous volume conduction, common drive, signal routing via parallel but disjoint paths, and the presence of cyclic paths within a network. All pose potential confounds for the metrics described here. The failure of Granger causality estimators in the presence of large amounts of measurement noise is a well-established shortcoming (Newbold, 1978) which becomes particularly acute in noisy electrophysiological recordings (Nalatore et al., 2007). Differences in the recording gain between signals is also known to confound estimation of Granger causality, with the most commonly used estimator demonstrating bias towards favouring the strongest signal as the driver (Bastos and Schoffelen, 2016; Haufe et al., 2012). This property is likely to be a nuisance when investigating causation between multimodal signal sets such as in experiments involving simultaneous measurements of MEG and LFP where significant differences in recording gain are to be expected (Litvak et al., 2011).

Instantaneous mixing of the electromagnetic signals generated by distinct sources in the brain has long been known to make estimation of functional connectivity based on recordings such as the EEG difficult (Haufe et al., 2012; Nunez et al., 1997; Srinivasan et al., 2007). Common presynaptic drive produces correlations in pairs of output spike trains (Farmer et al., 1993), and in pairs of evoked potentials (Truccolo et al., 2002). This problem can lead to spurious estimates of directed connectivity if delays in the arrival of the common input induce lagged correlations between unconnected neurons or neuronal populations. When the common presynaptic input is measured, extensions of functional connectivity metrics built upon partial regressions (so called *conditioned* or *partialized* estimates) can be used to remove common input effects and, subsequently, remove the possibility of spurious inference of directed connectivity between neurones in receipt of lagged common input. Partial regression can be used with both NPD and NPG to reduce the influence of common drive. In the case of NPD, the authors introduced a multivariate extension that can be used to reduce the influence of common drive through partial regression of a third reference signal (Halliday et al., 2016). This method relies upon the reference signal substantially encapsulating the activity of the common drive. In the case that the recordings are incomplete representations of the propagating neural activity, the conditioning will only be partially effective. NPD and NPG conditioned on a third signal can also be used to infer connectivity patterns where two signals are correlated through interaction with an intermediary signal (West et al., 2018).

Statistical aspects of coherence estimation have been widely studied. Carter et al. (1973a) highlight that a coherence estimate constructed from averaging over *n* independent segments has an asymptotic standard deviation proportional to $n^{-0.5}$, suggesting that a large number of segments are required to obtain reliable estimates. Carter et al. (1973b) suggest a reasonable range for *n* is 32–64 segments. The directional decomposition in NPD is based on the use of time lag in a correlation or partial correlation function. As the number of lags is related to the segment length, using shorter segments may impact on the reliability of directional estimates as fewer time lags are available to infer directional information.

In this paper we will assess the performance of NPD’s ability to recover the connectomes from several simulated architectures and in the presence of the previously stated confounds. We compare the accuracy of connectivity estimation with NPD and NPG under these different conditions. Furthermore, we also test the efficacy of a multivariate extension of NPD, the conditioned NPD, as a means of testing for the effects of common drive and its ability to discriminate between parallel signal routing. The amount of data required for accurate estimation of connectivity will also be assessed. Finally, we bring the presented methods to the analysis of empirically recorded data from patients with Parkinson’s disease. Using an example recording, we examine how artificially imposed changes in the signals’ SNR and linear mixing can change the estimate of dFC made between signals recorded from the human cortex and basal-ganglia. Our primary goal is to verify the utility of the proposed measure in application to real-world neuroimaging data.

## 2 Methods

### 2.1 Approach

In this study we utilize spectral coherence for estimates of undirected FC, and NPD/NPG for estimates of dFC. We set up models of continuous neural signals with known connectivity architectures parameterized in MVAR coefficients. Confounds such as signal-to-noise and instantaneous mixing are then introduced following simulation of the MVAR process using an observation model. The analyses presented here start with the assumption that any exploratory analyses of the data are complete, including any artefact rejection and/or preprocessing and that one or more significant coherence estimates have been identified as a prerequisite for directional decomposition using NPD. Using coherence, we first establish the existence of coherent frequencies within the modelled data sets. Patterns of connectivity in the models are then recovered using the two dFC metrics (NPD and NPG). As connectivity in the models is known (by design) we analyse how the metrics perform at accurately recovering the known connectivity profiles. Finally, we look at the methods’ application to empirical data when used to estimate the directed functional connectivity between the basal ganglia and motor cortex in recordings made from a patient with Parkinson’s disease (PD).

### 2.2 Analysis software and data availability

Data were analysed using a set of custom scripts written in MATLAB R2017a (The MathWorks, Natick, MA, USA). Non-parametric directionality was implemented using the Neurospec toolbox (http://www.neurospec.org/). MVAR models were implemented using the BSMART toolbox (Cui et al., 2008). NPG calculations and spectral estimates were implemented in FieldTrip (Oostenveld et al., 2011b). All scripts for the analyses presented here can be found in a GitHub repository (https://github.com/twestWTCN/NPD_Validate). A full list of script dependencies, toolboxes used, their authors, and links to their original source code can be found in Appendix I. The example patient data used in this paper is anonymised and available upon request.

### 2.3 Functional connectivity

#### 2.3.1 Spectra and coherence

Spectral estimates were made using periodogram estimates utilizing Hanning tapers. Unless otherwise stated (see section 3.6 in which we investigate the role of data availability), data were divided into segments 2^8^ samples in length (~1.3 ​s ​at 200 ​Hz). We computed the magnitude-squared coherence via:

(1) $${\left|R_{YX}\left(\omega \right)\right|}^{2}=\frac{{\left|f_{YX}\left(\omega \right)\right|}^{2}}{f_{XX}\left(\omega \right)f_{YY}\left(\omega \right)}$$

where $f_{XX},\;f_{YY},f_{YX}$ are the *X* and *Y* autospectra and *XY* cross-spectrum respectively.

#### 2.3.2 Non-parametric directionality

Non-parametric directionality provides a model-free estimate of directional correlations within a system through the decomposition of the coherence into components separated by their lags yielding separate instantaneous, forward-lagging, and reverse-lagging spectra (Halliday, 2015). This is achieved using pre-whitening of the Fourier transforms. This acts to bring the spectral content of a signal closer to that of white noise, in this case using optimal pre-whitening with minimum mean squared error to compute the whitening filter. This procedure is equivalent to generating two new random processes which have spectra equal to 1 ​at all frequencies:

(2) $$f_{XX}^{w}\left(\omega \right)=1,f_{YY}^{w}\left(\omega \right)=1$$

The prewhitening step effectively eliminates the autocorrelation structure of the respective signals but retains bivariate correlations between them. The pre-whitening brings the denominator of the coherence, the product of the autospectra (a normalization factor) equal to 1. Thus, the coherence can be reduced to the magnitude squared of the minimum mean square error (MMSE) pre-whitened cross spectrum:

(3) $${\left|R_{YX}\left(\omega \right)\right|}^{2}={\left|f_{YX}^{w}\left(\omega \right)\right|}^{2}.$$

The overall scalar measure of dependence between *X* and *Y*, denoted as $R_{YX}^{2}$, is defined as the integral over the coherence in equation (1). In line with the previous literature, the notation here uses as $R_{YX}^{2}$ to indicate a scalar measure of overall dependence and ${\left|R_{YX}\left(\omega \right)\right|}^{2}$ to indicate coherence, a function of frequency. As the coherence loses all terms in the denominator, the equivalent cross-spectrum can then be transformed to the time domain to yield the time-domain correlation function:

(4) $$\rho_{YX}\left(\tau \right)=\frac{1}{2\pi }\int_{-\pi }^{+\pi }f_{YX}^{w}\left(\omega \right)e^{i\omega \tau }d\omega .$$

This measure can be decomposed (in the time domain) via Parseval’s theorem for any desired lag. We choose to separate into reverse, instantaneous, and forward components:

(5) $$R_{YX}^{2}=\underset{X\leftarrow Y}{\underset{︸}{\int_{\tau <0}^{0}{\left|\rho_{YX}\left(\tau \right)\right|}^{2}d\tau }}+\underset{X↔Y}{\underset{︸}{{\left|\rho_{YX}\left(0\right)\right|}^{2}}}+\underset{X\to Y}{\underset{︸}{\int_{0}^{\tau >0}{\left|\rho_{YX}\left(\tau \right)\right|}^{2}d\tau }}.$$

These components may be abbreviated to:

(6) $$R_{YX}^{2}=R_{YX,-}^{2}+R_{YX,0}^{2}+R_{YX,+}^{2}$$

where component $R_{YX,-}^{2}$ yields correlations in which X lags Y, $R_{YX,0}^{2}$ instantaneous correlations, and $R_{YX,+}^{2}$ correlations in which Y lags X. To create a set of frequency domain measures which decompose coherence into three directional components, the three terms in equation (6) are each Fourier transformed using the lag ranges in equation (5). This creates three frequency domain measures that capture reverse, zero-lag and forward directionality, respectively. Coherence is decomposed by direction using a ratio of the relative magnitude-squared values at each frequency as:

(7) $${\left|R_{YX}\left(\omega \right)\right|}^{2}={\left|R{’}_{YX,-}\left(\omega \right)\right|}^{2}+\;{\left|R{’}_{YX,0}\left(\omega \right)\right|}^{2}+\;{\left|R{’}_{YX,+}\left(\omega \right)\right|}^{2}.$$

The prime symbol on the RHS of equation (7) is used to indicate that these are not formal coherence measures, but represent one of three directional contributions (reverse, instantaneous and forward) to the coherence. Thus, from each component we can assess spectrally resolved directional interaction whilst accounting for the signals’ autocorrelation structure. For a full derivation of the NPD method and details of its algorithmic implementation please refer to Halliday et al. (2016).

#### 2.3.3 A multivariate extension – conditioned non-parametric directionality

In addition to bivariate NPD, we used a multivariate extension that allows the directional components of coherence to be conditioned upon a third signal (Halliday et al., 2016). The conditionalization of NPD is achieved through a partial regression of *X* and *Y* conditioned on *Z*. This analysis decomposes the partial coherence into the same three directional components: forward, reverse, and zero-lag. It can indicate if information in the bivariate interaction shares variance common to signals in other parts of the network. For example, the partial correlation between *X* and *Y* with *Z* as predictor can be used to determine if the flow of information from *X* → *Y* is independent of area *Z*, or whether the flow of information is *X* → *Z* → *Y*, in which case the partial coherence between *X* and *Y* with *Z* as predictor should be zero. The partial coherence can also be used to investigate if the flow of information is *Z* → *X* and *Z* → *Y*, or if it is *X* → *Y* → *Z* or *Z* → *X* → *Y*, or in the case of common input *Z* → *X and Y*, in which cases the partial coherence, and any directional components, should be zero.

The relationship between the squared coherence function ${\left|R_{XY}\left(\omega \right)\right|}^{2}$ and the squared correlation coefficient was the starting point for the derivation of the non-parametric directionality method in Halliday (2015). The correlation coefficient is given by:

(8) $$R_{YX}^{2}=\frac{\sigma_{Y}^{2}-\sigma_{Y|X}^{2}}{\sigma_{Y}^{2}}$$

where the conditioned variance, $\sigma_{Y|X}^{2}$ is the variance of the error process following a linear regression of Y on X. It then follows that the correlation coefficient may be conditioned to account for any common effect that a process Z may have on both X and Y by also estimating the residuals following regression with Z:

(9) $$R_{YX|Z}^{2}=\frac{\sigma_{Y|Z}^{2}-\sigma_{Y|X,Z}^{2}}{\sigma_{Y|Z}^{2}}$$

in which the processes X and Y are both conditioned (regressed) against the third process Z. Partial regression is often useful in situations in which it is believed that the tertiary signal Z can account for some or all of the original association between X and Y. Thus, the objective is to distinguish whether there is a genuine correlation $R_{YX}^{2}$ that is distinct from the apparent one induced by Z: $R_{YX|Z}^{2}$. In the same manner by which the correlation coefficient may be conditioned to account for any common effect that a process Z may have on both X and Y, we can condition the estimated coherence ${\left|R_{YX}\left(\omega \right)\right|}^{2}$ on Z:

(10) $${\left|R_{YX|Z}(\omega )\right|}^{2}=\frac{{\left|f_{YX|Z}(\omega )\right|}^{2}}{f_{XX|Z}(\omega )f_{YY|Z}(\omega )}$$

In this way we can form a so called ‘partial’ coherence to determine the association of the coherence between X and Y with predictor Z. By using this form of the coherence as the starting step we can continue with the same decomposition as was made before for bivariate NPD, in order to attain an estimate of the NPD between X and Y but conditioned on Z. In practice we achieve conditioning of the respective autospectra $f_{XX|Z}\left(\omega \right)$ and $f_{YY|Z}\left(\omega \right)$ using the approach set out in Brillinger (1988). This method has been used successfully in LFP recordings to recover known anatomical pathways in the basal-ganglia (West et al., 2018). For full details of the derivation and implementation of conditioned NPD, see Halliday et al. (2016).

Increased levels of additive noise can impact on partial coherence estimates but should not distort any temporal precedence present in the triplets of signals (Baccalá and Sameshima, 2006). Conditioned NPD uses decomposition by time lag to infer directionality so should be robust to increased levels of additive noise in the predictor. We explore the extent to which this is true in simulations of triadic networks in results section 3.8.

#### 2.3.4 Non-parametric Granger Causality and its relation to NPD

Granger causality is based on the premise that if a signal *X* causes a signal *Y*, then the past values of *X* can be used to predict the state of *Y* beyond that of the information contained in the past of *Y* alone (Granger, 1969). This has conventionally been tested in the context of multivariate autoregressive models fit to the data, and in which the explained variance of *Y* via a ‘restricted’ model based on *Y* alone is compared to that of a ‘full’ model using information of both the past of *X* and *Y* (Geweke, 1982). Frequency domain extensions of Granger have been developed (Geweke, 1982; Kamiński et al., 2001) and applied widely across many domains of neuroscience (e.g. Brovelli et al., 2004).

The requirement to fit multiple MVAR models can cause several difficulties in analyses, namely: i) the requirement of large model orders to capture complex spectral features; ii) computational cost of model inversion; and iii) assumptions as to the correlation structure of the data in order to capture the signal as an MVAR process. In order to avoid the requirement for the estimation of MVAR models, Dhamala et al. (2008b) proposed a non-parametric estimator of Granger Causality. This estimator can be derived from widely used Fourier or wavelet based spectral estimation methods which do not suffer from these complications. The method hinges on the derivation of a spectral matrix directly from the spectral transforms of the data (i.e. Fourier or wavelet) instead of the full transfer and noise covariance matrices specified in an inverted MVAR model. Subsequently, the spectral matrix is factorized to derive the transfer function and noise covariance matrices of the set of signals (Sayed and Kailath, 2001). Via this technique it is possible to decompose the total power spectrum of Y between its intrinsic power and the *causal* contribution from X. The first term refers to the *intrinsic* power of Y, the second term to a causal contribution to the power of Y from X. For a full derivation and details of its implementation please refer to Dhamala et al. (2008b).

The difference between the way NPG and NPD determine causal or directional components is that NPG uses a decomposition of the signal power into intrinsic and extrinsic components, whereas NPD decomposes a normalised correlation coefficient according to time lag. Both NPG and NPD use a frequency domain approach. The frequency approach in NPG uses the formulation in Geweke (1982) in combination with factorization of the spectral matrix (Wilson, 1972), see Dhamala et al. (2008a, Dhamala et al., 2008 for details. NPD is based on the approach of Pierce (1979) to decompose the product moment correlation coefficient and coherence summatively into directional components. The starting point is the spectral matrix (as in NPG). The decomposition is achieved by generating an MMSE pre-whitened spectral matrix, $F_{w},$ as:

(11) $$F_{w}=\left(\begin{array}{cc} 1 & f_{YX}{\left(f_{XX}f_{YY}\right)}^{-0.5}\\ f_{XY}{\left(f_{XX}f_{YY}\right)}^{-0.5} & 1 \end{array}\right),$$

where $f_{YY}$ and $f_{XX}$ are the autospectra, and $f_{YX}$ and $f_{XY}$ are the cross spectra, with frequency argument omitted. The effect of this pre-whitening allows coherence to be calculated directly from the cross-spectra. NPD thus decomposes coherence according to time lag in the normalised correlation whereas NPG decomposes the spectrum into intrinsic and extrinsic factors, the presence of non-zero intrinsic factors is taken as indicative of a causal effect in NPG.

#### 2.3.5 Pairwise versus multivariate applications of metrics

Both NPD and NPG can be used in either a bivariate (pairwise) or a full multivariate (i.e. considering greater than two signals) framework. As pairwise analyses of dFC are by far the most common approach used in the current literature we primarily make a comparison of bivariate NPD and NPG computed between two signals only. However, when investigating issues such as common drive and the influence of tertiary signals we utilize the multivariate extension of NPG (mvNPG; Wen et al., 2013) and compare it with conditioned NPD. mvNPG extends Geweke’s formulation of Granger beyond pairwise analyses using spectral matrix factorization. In combination with Dhamala’s approach to obtain spectral matrices from Fourier transforms of data this yields a method by which it is possible to create a non-parametric estimator of causality in high dimensional data. mvNPG used here is implemented in Fieldtrip (Oostenveld et al., 2011a).

Conditioned NPD is indicated by the use of brackets to signify the conditioning signal (e.g. NPD(x) signifies NPD conditioned on signal X). This approach is used exclusively in section 3.1 (common drive) and 3.8 (incomplete signals for conditioning).

### 2.4 Generation of synthetic data

#### 2.4.1 Multivariate autoregressive modelling

In order to simulate data that describes a lagged propagation between simple periodic systems we used an MVAR modelling framework. MVAR models are an extension to 1-dimensional autoregressive models in which a model variable can be expressed as a linear combination of its previous values plus some stochastic error term. A *P*^*th*^ order MVAR model with N number of states is given by:

(12) $$X_{t}=c+\sum_{i=1}^{P}{\text{A}}_{i}X_{t-i}+\epsilon_{t}$$

where $X_{t}$ is a [N×1] vector of values at time *t*, and $X_{t-i}$ are the values at time (t−i).$\;{\text{A}}_{1},\ldots ,{\text{A}}_{P}$ are [N×N] matrices of autoregressive coefficients at lag i, c is a vector of constants, and $\varepsilon_{t}$ is innovation noise (Gaussian) with zero mean and covariance R. An AR model of order *P* describes the *N* values at time, $X_{t}$, as a linear combination of *P* previous values, $X_{t-i}$, a set of constants, *c*, and a vector of additional noise values, $\varepsilon_{t}$. The *P* matrices $A_{i}\;(i=1,\ldots ,P$) specify the linear dependencies in the model at time lag *i*. Simple periodic signals may be engineered in the MVAR formulation by setting of alternating signed coefficients at different lags. For example, to obtain a lag two periodicity of the system variable *X* we set ${\text{A}}_{1,2}=\left[1-1\right]$. The alternating signs of the coefficients set up the signal to oscillate with a period equal to the difference in lags. In order to introduce correlations between variables we introduced non-zero coefficients off the diagonal. In this way we simulate lagged connectivity by setting positive coefficients between nodes at lags greater than 1. For the parameters of the simulated MVAR models please see appendix II. Simulations were made using the BSMART toolbox. All simulations were run with sample length *T*, where *T* ​= ​5 x 10^4^ data samples (except in sets of simulations investigating data availability and benchmarking; see below). In order to set a time scale of the simulations we chose an arbitrary sampling frequency of 200 ​Hz which places simulations around the frequencies typically observed in neural data. This yields total simulation time of 250s (unless otherwise stated). The model architecture for each set of figures is outlined using a ball and stick diagram next to the main results. All MVAR models used were tested for asymptotic stability by determining that the absolute value of the eigenvalues of a model’s companion matrix were less than 1 (as per Lütkepohl, 2005).

#### 2.4.2 Observation modelling

To introduce the effects of changes in SNR and instantaneous mixing of signals that can arise due to the practical aspects of experimental recordings of neural signals, we construct an observation model on top of the model of the dynamics that maps from the hidden internal variables X onto the externally observed variables Y. This function adds observation noise to the MVAR signal and then applies an instantaneous linear combination of the internal variables:

(13) Y=z(LX)+λγ

where Y is a vector of observations created using the vector of internal variables, X, combined with a vector of additive observation noise (i.i.d, zero-mean, unit-variance, white noise) γ weighted by scalar factor λ which determines the effective SNR of the observed variables. The function z(⋅) indicates z-standardization to zero mean and unit variance. A [N×N] mixing matrix *L* is used to introduce dependencies between the observed signals. There is a constraint on the diagonal of *L* such that $L_{i=j}=1$, i=1,…,N such that the gain of the signals themselves was unaltered. Thus, we specify the mixing between signals by specifying the off-diagonal entries of matrix *L.* When applied to z-normalised, uncorrelated data, the mixing matrix introduces shared variance equal to the square-root of the off-diagonal coefficients of *L* (Halliday et al., 2016)*.*

We compute the decibel SNR as the log ratio of signal variances i.e. 1:1 SNR is equivalent to 0 ​dB. In some simulations we investigate the role of asymmetric SNR and so report the difference of SNRs between signals: $\text{Δ}SNR_{XY}\;=SNR_{X\left(dB\right)}-SNR_{Y\left(dB\right)}$. Assuming one signal is held constant then a difference in SNRs of 10 ​dB is equivalent to 10 times increase in the noise in the other signal, 20 ​dB equivalent to 100 times increase, etc. SNR calculations are computed from the ratios of the mean narrowband power within the range of the peak frequency of activities ±5 ​Hz with that of the background noise, providing good coverage over the example signals used here. In empirical neuroimaging data where multiple sources of noise exist, this is a much harder quantity to estimate (Parkkonen, 2010). We however use +12 ​dB as the level for the weakest signal, equivalent to a good quality EEG recording (Goldenholz et al., 2009).

### 2.5 Benchmarking the metrics: data length, number of connections, and combined confounders

To determine the quantity of data required to use either NPD or NPG for the accurate estimation of dFC, we setup a benchmark test and then examine how the score of this benchmark changes with the amount of available data and number of connections in a given network. We also use this benchmark to assess how a combination of confounding effects can influence network estimation. First, we randomly simulate three sets of 24 random directed graphs with a fixed number of vertices (*n* ​= ​3) and including either one, two, or three edges in total. These graphs are then simulated as MVAR models (as detailed above) by placing non-zero autoregressive coefficients with a random lag uniformly distributed in the interval [1 3]. The simulated data is then analysed with NPD and NPG. By using a non-parametric permutation test to form confidence intervals (see section 2.7.1 below) for each measure we determine the detection of a directed connection if 10% of the spectra for the given pair of nodes is over the 99.99% confidence limit to yield a predicted adjacency matrix $\hat{M}$. For every element of $\hat{M}$ that is equal to that in the actual adjacency matrix M (i.e. a true positive or negative) the score is +1; for every non-equal element (i.e. false positive or negative) the score is −1. Thus, the maximum score in a three-node network is +6 (all correct) and the minimum is −6. We report scores as percentages of the maximum from −100% to 100%.

In the first set of benchmarks we investigate both the role of data availability and the number of connections in a graph. We perform the benchmark with the 24 random graphs using: i) a fixed amount of data (500s), but variable trial length (2^3^ to 2^10^) (such as is the case when deciding how to epoch data from a ‘steady-state’); and ii) a variable amount of data but a fixed number of trials (n ​= ​100) (such is the case when analysing an event related study with a set number of repetitions).

In the second set of benchmarks we investigate how the combination of asymmetric SNR and signal mixing effects act to confound connectivity estimation. We make a 12 x 12 design, adding noise to the target node to achieve a range of narrowband (50–60 ​Hz) $\text{Δ}SNR_{1,2}$ of −45 dB–0 ​dB and then adding signal mixing to achieve 0%–100% shared variance. The weakest signal is again clamped to 12 ​dB. Note that the adjustment of SNR is done before signal mixing. The benchmark is then applied for data simulated from the 24 random graphs described above.

### 2.6 Experimental data

#### 2.6.1 Experimental protocol

In the final experiment of this paper, we investigate how the two dFC metrics (NPD and NPG) perform when estimating the dFC between the cerebral cortex (supplementary motor area; SMA) and the subthalamic nucleus (STN). This connection has been reported to be predominantly cortically leading in patients with Parkinsonism (as estimated with NPG in Litvak et al., 2011, 2012). In this paper we use an example recording in which NPG reveals a clear directed component from SMA → STN. This recording was taken from a cohort of patients with PD who have undergone surgery for deep brain stimulation (DBS). The experimental data contains recordings made using whole head MEG and simultaneous LFP recordings from DBS electrodes implanted into the STN. The recordings were made for approximately 3 ​min with the patient quietly at rest with their eyes open. Experiments investigated the differences in MEG and LFP activity and connectivity when patients were withdrawn from their usual dose of L-DOPA (OFF) versus the L-DOPA treated state (ON). Patients were not undergoing stimulation with DBS at the time of the recording. The two-time series analysed were 183s in duration with MEG from a right SMA virtual sensor recorded simultaneously with an LFP from the right STN in a PD patient in the OFF state at rest. All experiments were conducted in a study approved by the joint ethics committee of the National Hospital of Neurology and Neurosurgery and the University College London Institute of Neurology. The patient gave their written informed consent. For full details of the surgery, implantation, recording, and experimental paradigm please see Litvak et al., (2011).

#### 2.6.2 Preprocessing

The MEG and LFP signals were first down-sampled to 200 ​Hz. They were then preprocessed using a high-pass filter (passband at 4 ​Hz, finite impulse response, two-pass, filter order optimized for data length). Recordings were truncated 1s at either end to remove border artefacts arising due to movement and equipment initialization. Finally, data were visually inspected to determine the presence of large abnormalities and high amplitude transients. In the case of the example data used here, none were found.

We performed estimation of the empirical SNR of the signals as detailed in section 2.4.2. The empirical data was first standardized to unit variance and then spectral peaks in the 14–31 ​Hz were compared with that of a white noise surrogate also with unit variance. The ratio between the peaks is reported as the estimated empirical SNR, equivalent to the difference of the spectral peak (in beta band) with that of the noise floor.

Changes to the SNR, asymmetric SNR, and linear mixing of the empirically derived signals were introduced using the same process as listed in section 2.4.2. This treatment ignores the fact that the data by necessity of empirical recording have already undergone observation with a transform similar in form to that in equation (13) but with unknown parameters regarding the lead-field (mixing matrix) and observation noise. Instead we take the empirical recordings as a ground-truth and investigate subsequent changes following artificially induced confounds.

### 2.7 Statistics

#### 2.7.1 Permutation confidence intervals

In order to form confidence intervals for the connectivity metrics we make no assumptions as to the form of their distributions but instead form permutation distributions of the metrics estimated from surrogate data and computed using a non-parametric rank order significance threshold (Theiler et al., 1992). We adopt a phase randomization approach to generate surrogates (Breakspear and Terry, 2002; Lancaster et al., 2018) which acts to preserve the magnitudes of the spectral estimates whilst scrambling the phase and hence disrupting any interaction between signals. For details and a discussion of the algorithm please see appendix I as well as Lancaster et al. (2018). For each test we generated 1000 realizations of the surrogate process. We obtain the P ​= ​0.001 confidence limit by taking the 99.9th percentile of the resulting distribution. Limits are plot in figures as a dashed line with arrows on the side of the axis to indicate their values.

#### 2.7.2 Least-squares regression

In the case of some confounds the response profiles were found to be sigmoidal functions with a maximum response, midpoint $x_{0}$; and steepness κ. We used a least-squares regression to fit the logistic function. All reported fits exceeded R^2^ ​> ​0.95 and we report the estimated parameters of the curves as summary statistics of the connectivity metrics’ modulation by a confound.

## 3 Results

### 3.1 Organization of the results

**Fig. 1:**
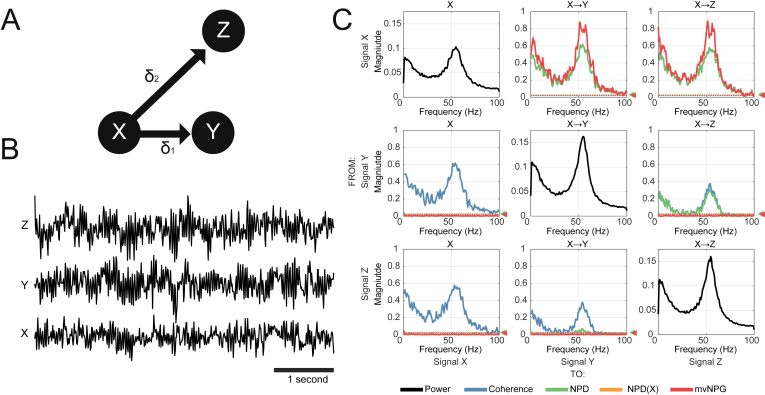
**Three -node simulation of MVAR model to compare functional connectivity measures**. **(A)** A simple three state, 3rd order MVAR model was used to simulate coupling of autonomous periodic signals. Connectivity was simulated using non-zero coefficients at lag 2 for node X → Y, and lag 3 for X → Z. Correlations are lagged such that the time delays are unequal (i.e. δ_1_ ​< ​δ_2_)**. (B)** Example 5-s realization of the simulated MVAR processes. **(C)** Connectivity matrix of the coupled signals. Autospectra are shown on the diagonal (black). Undirected functional connectivity (coherence) is shown in blue. Estimates of directed connectivity are shown for multivariate non-parametric Granger causality (mvNPG; red); Non-parametric Directionality (NPD; green); and NPD conditioned on signal X (NPD(X); orange). NPD identifies spurious directional connectivity between Y and Z due to the lagged correlations of X → Y relative to X → Z. Spurious connectivity is removed partializing the NPD estimate upon the signal at the common source at node X (NPD(X)) which acts to remove all spurious connectivity. Permutation confidence intervals (P ​= ​0.001) are shown for NPD and mvNPG by the green and red dashed lines and arrows respectively.

**Fig. 2:**
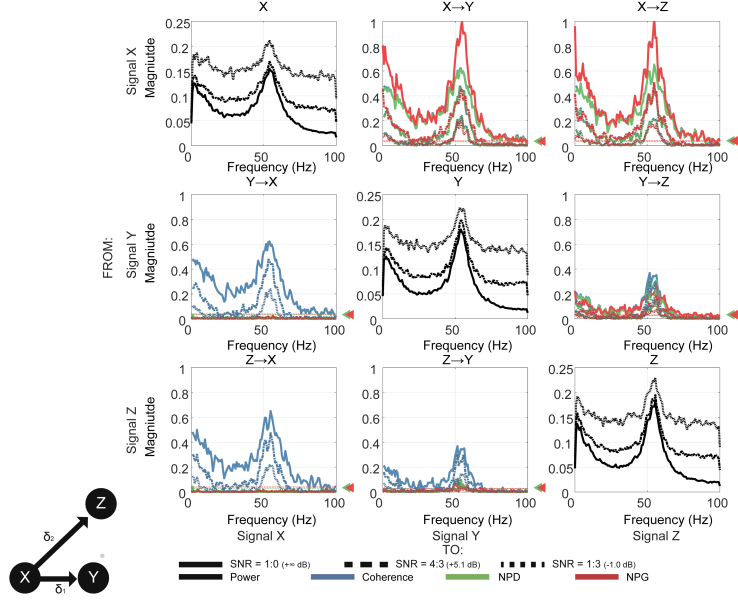
**Analysis of the effects of signal-to-noise ratio (SNR) upon estimators of directed functional connectivity.** The confounding effects of poor SNR were simulated by adding Gaussian noise to the MVAR processes and standardizing the overall variance equal to 1. The MVAR model is identical in form to that used in Fig. 1 and its structure is given by the ball and stick diagram in the inset. Simulated narrowband (45–55 ​Hz) SNRs at: 1:0 (+∞ dB; bold), 4:3 (+5.3 ​dB; ---), and 1:3 (- 1.0 ​dB; ···). The effects upon coherence (blue), NPD (green), and non-parametric Granger causality (NPG) were investigated. All estimators were reduced by increased levels of noise. Permutation confidence intervals (P ​= ​0.001) are shown for NPD and NPG by the green and red dashed lines and arrows respectively.

**Fig. 3:**
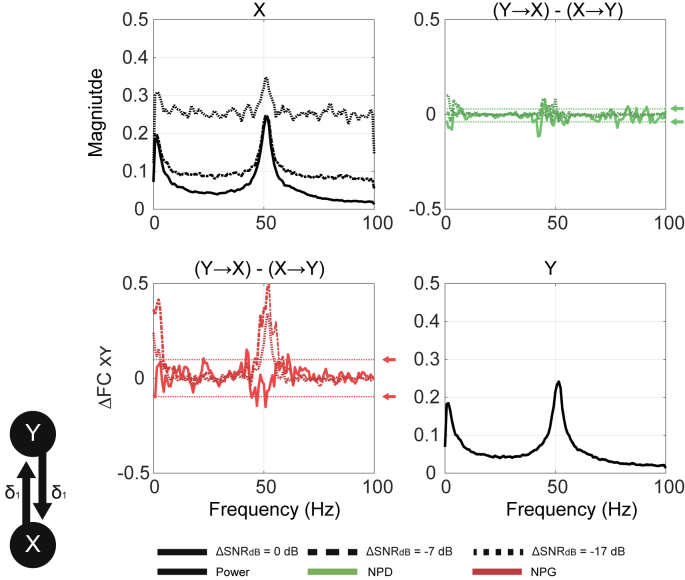
**Analysis of the effects of unequal signal-to-noise ratios, measured as a difference of the SNRs between X and Y (**$\Delta \mathrm{SN}R_{\mathrm{XY}}$**) upon symmetrical directed functional connectivity (dFC)**. The confounding effect of connected signals having different SNRs was simulated by the addition of Gaussian noise to signal X but fixing the noise of node Y to yield +13 ​dB. A range of differences in SNR between X and Y ($\text{Δ}SNR_{XY}$) were simulated at 0 ​dB (bold), - 7 ​dB (---), and - 17 ​dB (···). Connectivity was held fixed to be symmetrical. We assessed dFC by plotting the difference in magnitudes of the connectivity for each direction ($\text{Δ}FC_{XY}$) with $\text{Δ}FC_{XY}$ ≈ 0 as the ground truth. Results from both non-parametric directionality (NPD; green) and non-parametric Granger causality (NPG; red) are shown. In the face of medium amounts of SNR asymmetry, NPG spuriously identifies the strongest signal as the driving node. NPD suffers less from this issue and yields approximately symmetrical estimates for all conditions tested.

**Fig. 4:**
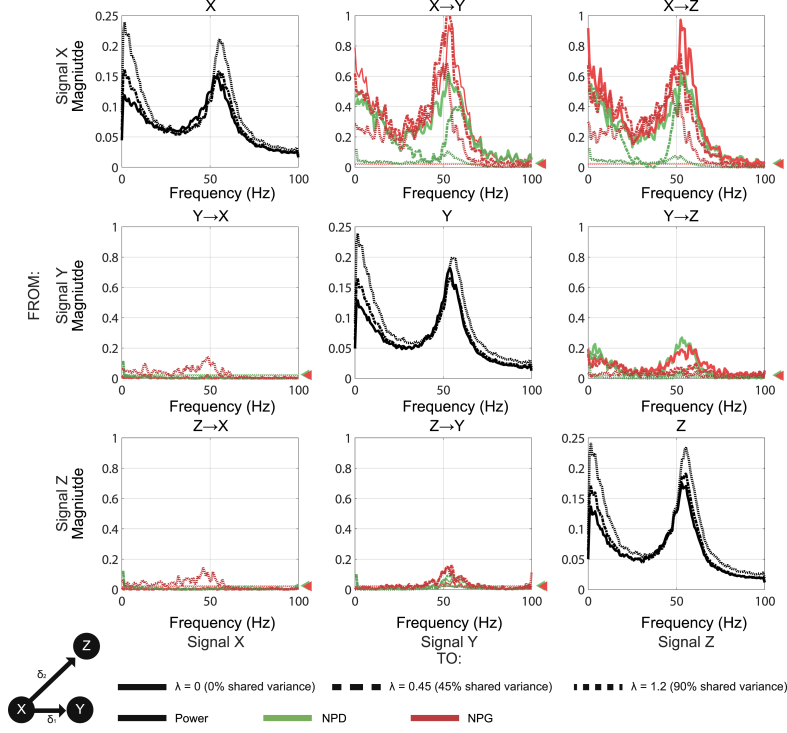
**Analysis of the effects of instantaneous mixing upon estimates of directed functional connectivity (dFC).** The confounding effects of volume conduction were simulated by multiplication of signals with a mixing matrix with off-diagonal coefficients *λ*. The unmixed signals were first generated with a three state, 3rd order MVAR model (identical to that used in Fig. 1, Fig. 2). We simulate three mixing conditions: *λ* ​= ​0 (zero mixing; bold line), *λ* ​= ​0.45 (45% shared variance; ---), and *λ* ​= ​1.2 (90% shared variance; ···). dFC is estimated using the lagged components of the NPD (green) or non-parametric Granger (NPG) (red). Permutation confidence intervals (P ​= ​0.001) are shown for NPD and NPG by the green and red dashed lines and arrows respectively.

**Fig. 5:**
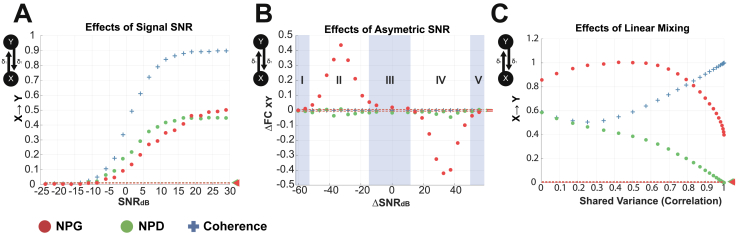
**Investigating the effects of signal-to-noise ratios (SNR), SNR asymmetries, and instantaneous linear mixing upon functional connectivity measures: coherence (blue), non-parametric directionality (NPD; green), and non-parametric Granger causality (NPG; red).** All measures are reported as the peak value for each individual estimate. Permutation confidence intervals (P ​= ​0.001) are shown for NPD and NPG by the green and red dashed lines and arrows respectively. **(A)** The effect of SNR was tested in the range from −30 dB to +30 ​dB. All estimators were found to have a sigmoidal response, with half-maximal suppression around SNR ​= ​0 ​dB. **(B)** The effect of unequal SNR between nodes X and Y ($\text{Δ}SNR_{XY}$) was varied by addition of observation noise to node X or Y separately to yield a range of narrowband $\text{Δ}SNR_{XY}$ from −60 dB to ​+ ​60 ​dB whilst coupling strengths were held fixed. NPG incorrectly identifies asymmetrical coupling for a wide range of $\text{Δ}SNR_{XY}$ (within zone II from −50 dB to −10 dB as well as zone IV from +10 ​dB to 50 ​dB). NPD estimates a weak bias towards one signal leading but with differences in directionality remaining close to zero across the range examined. **(C)** The effect of instantaneous signal mixing was examined across a range of mixing coefficients (*λ*) to yield a range of 0%–100% shared variance. Coherence is shown to increase as zero-lag correlations predominate with increasing valued *λ*. The lagged NPD shrinks to zero as instantaneous component of coherence dominates. NPG increases to a maximum at around 65% signal mixing and then sharply falls to zero. Permutation confidence intervals (P ​= ​0.001) are shown for NPD and NPG by the green and red dashed lines and arrows respectively.

**Fig. 6:**
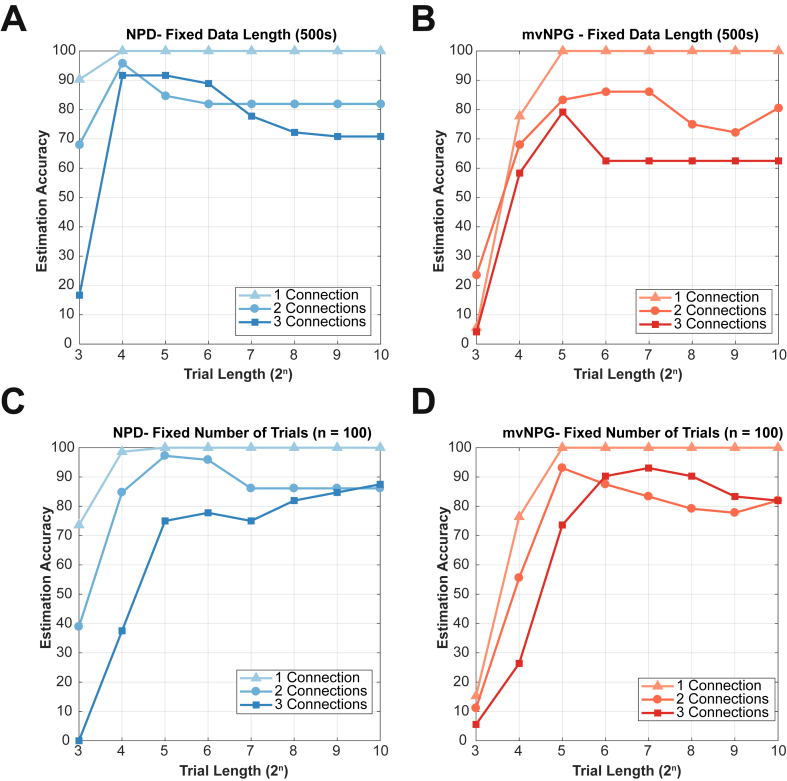
**Investigating the role of data availability upon the accuracy of connectivity recovery when using non-parametric directionality (NPD; blue), and non-parametric Granger causality (NPG; red).** The two estimators were benchmarked against three sets of 24 MVAR models with random connectivity comprising either one, two, or three connections respectively. Different amounts of data were simulated for each model and the accuracy of the recovery was scored using the criteria set out in the methods. Simulated data is sampled at 200 ​Hz. **(A and B)** Benchmarks recorded from analyses of simulated data in which there was a fixed amount of data (500s) but allowing for variable trial lengths (in samples). **(C and D)** Benchmarks recorded from analyses of simulated data in which there were a fixed number of trials (n ​= ​100) but variable total data length.

**Fig. 7:**
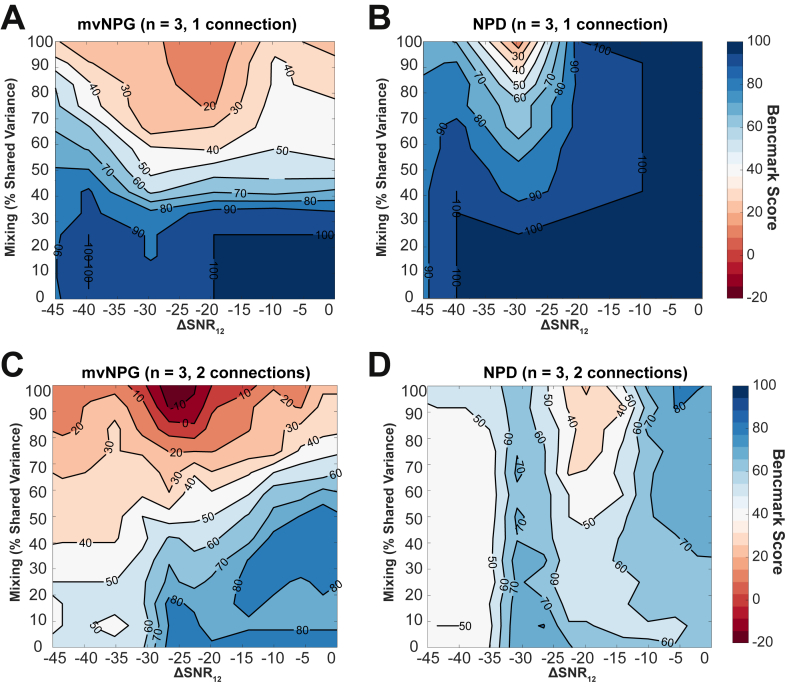
**Investigating the role of combined data confounds (instantaneous mixing and asymmetric signal-to-noise ratio;**$\Delta \mathrm{SN}R_{\mathrm{XY}}$**) upon the accuracy of connectivity estimation when using non-parametric directionality, and non-parametric multivariate Granger causality.** The two measures were benchmarked against two sets of 24 realizations of a 3-node MVAR models comprising either one (top row), or two (bottom row) randomly placed connections. For each simulation 200s of data was simulated and divided into epochs of 2^8^ samples. Simulated data is sampled at 200 ​Hz. All data is represented as a contour plot when varying first instantaneous mixing from 0% to 100% shared variance; and then adjusting the approximate asymmetric narrowband (45–55 ​Hz) SNR from −45 dB to 0 ​dB. **(A)** Benchmarking of mvNPG with simulations containing one randomly placed connection on a three-node network. **(B)** Same as for (A) but using NPD. **(C)** Benchmarking of mvNPG with simulations containing two randomly placed connections on a three-node network. **(D)** Same as for (C) but using NPD as the estimator.

In the following section the effects of common drive, degradation of SNR, asymmetric SNR, instantaneous signal mixing, data availability, and simultaneous confounders upon estimation of dFC using NPD and NPG are investigated. In Fig. 1, Fig. 2, Fig. 3, Fig. 4 examples of the impact of these individual confounding factors upon the power and connectivity spectra are presented. In order to summarise the effects of the confounds across a much larger range of scales, in Fig. 5 the effects of SNR, unequal SNR, and signal mixing are visualized as a plot of the relevant statistic of the connectivity (i.e. strength or asymmetry) against a scale of values of the confounding factor. In each of the following sections (3.2–3.5) we inspect first the example spectra displayed in Fig. 1, Fig. 2, Fig. 3, Fig. 4, and then go on to establish the total effect over the full range of the confound using the data illustrated in Fig. 5. Fig. 6, Fig. 7 use a benchmarking approach to quantify the accuracy of recovery given differing data lengths and mixed confounds. In the final section and Fig. 9 we look at application of the metrics to empirically recorded data.

### 3.2 Effects of lagged dependencies and common drive

We first demonstrate the efficacy of the metrics at recovering simple hierarchical architectures and establish how common input can act to confound them. To this end we present results from a simple 3-state, 3rd order MVAR model with no signal mixing and zero observation noise. The MVAR model is imbued with periodic dynamics that are identical at each node and are driven by noise with fixed covariance structure. Non-zero (off-diagonal) matrix coefficients are all fixed at 0.5 and the full MVAR parameters can be seen in table 1 of appendix II. We design the MVAR model (Fig. 1A) such that all edges originate at node X and correlations are lagged such that an input arriving at node Z lags that at node Y (δ_1_ ​< ​δ_2_). This introduces a deliberate confound as dFC methods estimating causality in a way dependent upon temporal lag will assign spurious causality from Y to Z, due to the difference in arrival times of input from X. An example time series of the process is shown in Fig. 1B and the resulting analyses of the functional connectivity are shown in Fig. 1C.

This model generates rhythmic activity at ~55 ​Hz as indicated by the peaked autospectrum for each node. Functional connectivity as measured using standard coherence shows significant connectivity (>0.5) between all nodes, albeit reduced for the connection between Y and Z. We next estimate directed connectivity using NPD. NPD shows that all connections are in the forward direction for X → Y and X → Z. As the full coherence is equal to the sum of the directional components, the overlap of the forward NPD (spectra in the top-right panel of the figure) with the coherence shows that they are equivalent in this case. The shorter lag in transmission from node X → Y compared to X → Z, results in a spurious estimate of coupling from Y → Z when estimated with NPD. However, when we condition the NPD upon the signal that both node X and Z receive common input from (NPD conditioned on node X; NPD(X)), we see that the Y→ Z correlation is abolished.

When pairwise Granger (NPG) was applied to the simulated data, the connectivity from X to Z and Y was very similar in form to the unconditioned NPD (data not shown). However, as the multivariate estimator of Granger (mvNPG) considers the full covariance across all nodes, its application acted to remove the spurious Y→ Z correlation that arose due to the common drive. This limitation of pairwise NPD is readily overcome using the multivariate extension that allows for the conditioning of the common input. In this way the results between mvNPG and NPD conditioned upon the common input (NPD(X)) are comparable. Both NPD(X) and mvNPG give estimates of Y → Z that are below the P ​= ​0.001 confidence interval indicating the absence of any significant directed connectivity between X and Y.

### 3.3 Effects of low signal-to-noise ratios

Recordings of field activity in the brain are made in the presence of both endogenous neural background activity as well as observer noise originating from recording equipment and other sources outside the brain. In Fig. 2 we simulate the effects of signal-to-noise ratio (SNR) upon estimates of functional connectivity. The variance of the MVAR process was standardized and so was equal in all simulations. We used additive Gaussian noise in the observation model to simulate an SNR of 1:0 (+∞ dB), 4:3 (+5.3 ​dB), and 1:3 (- 1.0 ​dB). All functional connectivity metrics were resistant up to moderate amount of additive noise (SNR_dB_ ​= ​+ 5.3 ​dB), but all estimates were heavily attenuated for the greatest noise tested in Fig. 2 i.e. SNR ​= ​−1.0 ​dB. When looking across a wider range of SNRs (Fig. 5A), both NPD and NPG approached 0 when the data became almost entirely noise i.e. SNR approached 0:1 (- ∞ dB). Responses were sigmoidal for all three metrics measured with half maximum suppression around 50% signal loss. Non-linear least-squares fitting yielded parameter estimates of the logistic rise for each FC estimator (midpoint $x_{0}$ and steepness κ): coherence $x_{0}^{Coh}$ ​= ​1.12 ​dB, $\kappa^{coh}$ ​= ​0.12; NPD $x_{0}^{NPD}$ ​= ​1.47 ​dB, $\kappa^{NPD}$ ​= ​0.11; and NPG $x_{0}^{NPG}$ ​= ​+ 7.1 ​dB, $\kappa^{NPG}$ ​= ​0.07. From these estimates and the curves shown in Fig. 5A, it is clear that coherence and NPD effectively share the same response profile to SNR. NPG is more sensitive to noise with estimates becoming degraded at higher SNRs ($x_{0}^{NPG}>\;x_{0}^{NPD}$). Overall the two metrics have a difference in the midpoints of the calibration curves of ~8 ​dB, with NPG being more sensitive to noise by almost an order of magnitude greater than NPD. However, the differences between the NPG estimator and NPD result in different SNR thresholds required to detect statistically significant connectivity (i.e. greater than the P ​= ​0.001 confidence threshold) and so the measures reach significance at different SNR levels: for NPG at −7 dB (SNR ​= ​1:5), and for NPD at −11.5 ​dB (SNR ​= ​1:14).

### 3.4 Effects of differences in signal-to-noise ratios between signals

Asymmetries in the SNR of different signals are known to distort the estimation of dFC when using methods based upon Granger causality (Bastos and Schoffelen, 2016; Nolte et al., 2008). We next tested whether this was true for NPD. We simplified the model to contain just 2 nodes that were reciprocally connected with the same lag. Again, the output of the MVAR model was standardized to have unit variance. We then modified the SNR of the first node (X) via the same process as for the previous set of simulations but fixing the variance of the noise of the second (Y) node to yield an SNR of +13 ​dB. Signals were constructed with a difference of SNRs between X and Y ($\text{Δ}SNR_{XY}$ ) equal to −17 dB, −7 ​dB, and 0 ​dB and calculated with respect to the SNR of Y which was held constant. The results of the simulations are shown in Fig. 3, Fig. 5B. In Fig. 3 we plot the difference in the estimates of the directed connection (i.e. X → Y minus Y → X; $\text{Δ}FC_{XY}$) to explore any deviation away from the symmetry in functional connectivity expected from the MVAR model ($\text{Δ}FC_{XY}$≈0).

Our simulations confirm that NPG is biased by differences in SNR between signals, showing that even at moderate asymmetries (i.e. at $\text{Δ}SNR_{XY}$ ​= ​−7 ​dB) the weaker signal is estimated to be driven by the stronger i.e. Y→ X. NPD suffers far less from this confound and maintains estimation of the difference in coupling as close to zero for all conditions tested. Analysis with NPD shows far less deviation from the ground truth of symmetrical coupling when the SNRs are unequal. When looking across a range of SNR asymmetries the response of each measure is apparent (Fig. 5B). NPG spuriously identifies directed coupling, with the bias for Y leading when $\text{Δ}SNR_{XY}$ is in the range −10 dB to −50 dB and peaking around −35 dB (zone II of Fig. 5B). In contrast, the bias for X leading is when X has a stronger SNR and $\text{Δ}SNR_{XY}$ is in range of +10 ​db to +50 ​dB, and peaking around $\text{Δ}SNR_{XY}$ ​= ​+35 ​dB (zone IV). At very large (positive) or very small (negative) $\text{Δ}SNR_{XY}$ the bias in coupling is diminished and there is a return to symmetrical estimate of connectivity as both NPD and NPG approach 0 for both directions (zones I and V). However, whilst NPD exhibits a much weaker bias than NPG it does still demonstrate an above significant difference in connectivity. However, deviations in estimation of symmetrical coupling arising due to unequal SNR are roughly an order of magnitude smaller than NPG with a maximum $\text{Δ}NPD_{XY}$ of −0.045 versus $\text{Δ}NPG_{XY}$ of −0.42. In terms of deviation from the difference in measures when $\text{Δ}SNR_{XY}$ ​= ​0 ​dB, NPD shows a maximum of 1.5 times inflation in asymmetry. For NPG this bias is a maximum of 20 times larger indicating its increased susceptibility to unbalanced SNRs.

### 3.5 Effects of instantaneous signal mixing

Neurophysiologically recorded signals such as MEG, EEG, and LFPs are subject to instantaneous mixing of the underlying dipole currents as a result of field spread effects. We next simulate these effects by multiplication of the simulated MVAR process with a linear mixing matrix and investigate the influence of mixing coefficients upon estimates of dFC. We use an identical model to that in section 3.2 (3 state, 3rd order MVAR) but with the addition of the observer model to model signal mixing at a range of values of *λ* to yield simulations with 0%, 20%, and 60% shared variance. There is no observation noise added. The results of the analysis are shown in Fig. 4.

The confounding effect of instantaneous mixing was established by first estimating the degree to which it may influence the symmetrical zero-lag component of the NPD. As expected, it was found that the zero-lag NPD is increased by mixing (data not shown), particularly at frequencies outside of the periodic component of the signal. This occurs as in the case of the unmixed signals, correlation between processes is dictated by off diagonal MVAR coefficients at lags greater than zero. When mixing is introduced, common noise from outside the main frequency band of interaction more readily overcomes the intrinsic noise at each node (which is weaker in power than activity at the interaction frequency) and so results in the largest zero-lag correlations outside of the main periodic component of the signals.

When using NPD to estimate dFC we found that it accurately reconstructs the designed connectivity up to a moderate degree of signal mixing (45% shared variance), albeit with a reduction of the estimated magnitude of connectivity (e.g. 0.6 to 0.4 for X → Y). At the highest degree of mixing (90% shared variance) the spurious connectivity between nodes Y and Z (introduced by the lagged common drive from node X) becomes increasingly symmetrical with an increase in the connectivity in the reverse direction (i.e. Y → X) despite the absence of these connections in the model arising either by design or by lagged common input. Overall, with increased signal mixing, the estimate of NPD is weakened equally across all connections. Analysis with NPG however shows that mixing has the effect of introducing spurious connectivity between Y and Z, exhibiting a small but significant reversal connectivity at Z → Y at even moderate mixing (45% shared variance). At the greatest degree of mixing, NPG determines statistically significant connections (i.e. above the P ​= ​0.001 permutation confidence interval) for Y → X and Z → X, neither of which are in the underlying model. Unlike NPD, which shows a uniform reduction in magnitude with increased mixing, the magnitude of NPG estimates depends upon the initial SNR of the nodes. In this instance the X → Z connection is weakened whilst the X → Y is strengthened. This effect is due to the process explored in section 3.4, by which unequal SNR biases the NPG estimator.

When testing across a wider range of degrees of signal mixing (Fig. 5C) the difference in the response of NPD and NPG is apparent. When using NPG to estimate dFC the magnitude of the estimate of X→ Y increases to a maximum at around 50% shared variance and then quickly collapses at very high mixing as instantaneous correlations begin to predominate. This result is related to the findings of section 3.4 in which it was shown that signal leakage acts to modify the effective SNR of the signals such that leakage from one signal can act to bias causality estimates towards another signal at even moderate amounts of instantaneous mixing. This effect is apparent when looking at the trace of the standard (symmetrical) coherence (in blue) which drops to a minimum at ~30% shared variance but then increases as zero-lag correlations take over. As the NPD explicitly ignores the zero-lag component, it displays simpler behaviour, and reduces in amplitude with increased mixing. This occurs because zero-lag coherence predominates, and the lagged components become increasingly small.

### 3.6 Effects of data availability upon benchmarks of the metrics’ accuracy

Application of functional connectivity metrics to real world data are often limited by the amount of data that is available to estimate them. In the next series of analyses, we quantify the dependency of accurate estimation upon the sufficiency of the given data. We use the benchmarking approach laid out in the methods and examine the role of data availability in two ways: 1) data which is continuous but of fixed total length (500s) data which is variable in length but with a fixed trial length. The results of this are shown in Fig. 6. In Fig. 6A and B we compare NPD with mvNPG in the accurate recovery of known patterns of connectivity using a benchmark score of accuracy (see method section 2.5) when using a fixed amount of data. We observe a common trend that the overall accuracy of recovery increases with trial length, reaching a maximum at the highest trial length tested at 2^10^ samples (equivalent to 39 trials each ~5s in duration assuming a 200 ​Hz sampling rate). The recovery of denser models required longer trial lengths and overall were estimated less accurately. This effect occurs due to the introduction of common drive effects inducing false-positive detection of connections. Sparser networks including just one single connection reached maximum accuracy with trial lengths as short as 2^5^ samples (equivalent to 1250 trials each ~0.16s in duration). With a fixed data length, it was found that NPD required shorter trials than mvNPG to reach similar degrees of accuracy (Fig. 6A versus 6B). Accuracy of recovery falls off with longer duration trials as the reduced number of repetitions hinders accurate estimation.

In Fig. 6C and D, the total data availability (i.e. 100 trials of variable duration) upon estimator accuracy was investigated. Again, both metrics displayed improved accuracy with increasing trials lengths. However, with longer trial lengths, mvNPG was able to accurately recover connectivity of models with denser connectivity than for NPD: in the case of networks with three random connections, and with a long trial length of 2^10^, mvNPG reached ~85% benchmark score versus 65% for NPD. Sparser models with just one or two connections showed an optimal trial length around 2^5^ (0.16 ​s ​at 200 ​Hz) for mvNPG versus 2^6^ (0.32 ​s ​at 200 ​Hz) for NPD.

### 3.7 Effects of combined confounds: instantaneous mixing and asymmetric signal-to-noise ratios

Empirically recorded signals are subject to several simultaneous confounding effects. In Fig. 7 we present results from a benchmarking analysis in which we confound simulated signals by introducing both asymmetric signal to noise, as well as instantaneous mixing of sources to a range of combined degrees. In Fig. 7A and B we compare the performance of mvNPG and NPD in the estimation of connectivity in MVAR models with one random connection. We vary instantaneous mixing from 0 to 100% shared variance; and asymmetric SNR at −45 dB–0 ​dB. These simulations show that when estimating connectivity using NPD in sparse networks with just a single connection there is a highly accurate recovery (>95% benchmark scores) unaffected by asymmetric SNR, and only with high shared variance (>50%) is there any significant drop in accuracy. A combination of strong asymmetry (−30 ​dB) and high mixing can however reduce benchmark scores to 30%. In comparison, mvNPG demonstrates poor accuracy across a much wider area of the conditions tested - with the benchmark reduced from 100% to 20% in the presence of negative asymmetric SNR greater than −20 dB (i.e. source node is weakest) and at all degrees of mixing above 20%. Benchmark scores are weakened further by the coincidence of strong instantaneous mixing of the signals.

In Fig. 7C and D we vary instantaneous mixing from 0 to 100% shared variance; and asymmetric SNR at −45 dB–0 ​dB. These tests show that mvNPG is more readily corrupted by both confounds with scores ranging from 80% to 50% along the asymmetric SNR axis, and from 80% to −10% along the axis in which instantaneous mixing is varied. Furthermore, the estimation of denser connectivity with NPD is hindered by asymmetric SNR but is less susceptible to combined signal mixing. Whereas the negative benchmark scores for mvNPG indicate common detection of false positives, scores for NPD do not drop below 40% for any of the combined confounds tested.

### 3.8 Confounds for conditioned directed connectivity arising from incomplete measurement of signals

We next investigate the properties of the multivariate extension to NPD which we term *conditioned* NPD. Conditioned dFC provides a more powerful method with which to explore network functional connectivity; however, in empirical cases, conditioning with a tertiary signal Z may not produce complete attenuation of the spuriously inferred directed connection between X and Y arising from the common input Z. This may arise as a result of: i) incomplete capture of the activity occurring at Z; and/or ii) difference in the routing of signals; and/or iii) because there are other sources of the spuriously inferred connection than Z alone. In cases where structural connectivity is well understood, and the conditioned signal Z is not expected to interconnect the path between nodes X and Y, any attenuation when conditioning can be assumed to arise in information propagated forward in the network (feedforward). On the other hand, if anatomical connectivity is unclear the effect of conditioning upon directed connectivity may also be explained by conventional serial routing (i.e. X → Z→ Y) but with incompleteness of observed signals at Z resulting in only partial attenuation of the X → Y estimate. In the next set of tests, we ask whether there are any differences in how the measures of dFC behave in the face of incomplete signal observation.

**Fig. 8:**
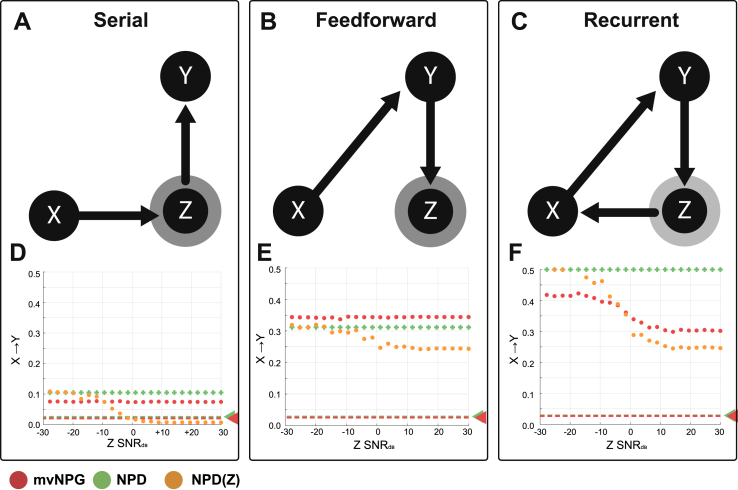
**The effects of incomplete signal observation upon estimation of directed functional connectivity: non-parametric Granger causality (NPG); non-parametric directionality (NPD); and NPD conditioned on reference signal Z (NPD(Z)).** Simulations investigate the connectivity of X → Y and the influence of propagation involving a tertiary node Z. We simulate incomplete sampling of Z by modifying its signal-to-noise ratio (SNR) via the addition of Gaussian white noise and then standardizing the variance equal to 1. **(A and D) Serial propagation –** signals propagate from X → Z → Y. The results of changing the SNR of Z are shown in panel D. Simulations demonstrate that dFC estimation with NPD/NPG are constant. At complete signal observation (SNR 1:0; +∞ dB), conditioning removes the estimate of dFC. With increasing SNR, the attenuation is diminished to the point where conditioning has no effect. **(B and E) Feedforward connectivity –** signals propagate to feedforward to the tertiary node: X → Y → Z. We find that conditioning has a weak effect (panel E), and the attenuation of NPD(Z) for estimation of X→ Y is again reduced by decreasing SNR of Z. **(C and F) Recurrent connectivity –** a further connection is added to the model to complete a cyclic path in the network: X → Z → Y → X. Decreasing the SNR of Z results in an increased estimation of NPG in X → Y (panel F). We again find that increased completeness of observation of Z results in an increase in the efficacy of NPD(Z) in determining tertiary (non-direct) signal routing.

For this set of simulations, we use a three state, 3rd order MVAR model, with all nodes generating identical autonomous dynamics and identical cross-node coefficients at equal model lags. We test three model connectivities to compare three types of signal propagation: a) serial (i.e. X → Z → Y); b) feedforward (i.e. X → Y → Z); or c) recurrent (i.e. X → Y → Z → X). We simulate incomplete observation of Z by modifying the SNR as was done in section 3.2. The model architectures and results of simulations are shown in Fig. 8. We demonstrate that in the simplest case of a serial path, the NPD conditioned on signal Z (NPD(Z)) behaves as expected: the estimate of connectivity X → Y is attenuated as all information between them is routed via Z. With decreasing SNR of the observation of Z we show that conditioning has less and less effect and converges to the estimate yielded by the unconditioned NPD. Pairwise NPD remains constant at all SNRs tested as it does not account for any of the activity at Z. In these simulations, multivariate NPG (mvNPG) was also applied as a way to estimate directed connectivity that accounts for all signals in the model. We find that mvNPG shows a small decrease (~0.025) in the estimate of X→ Y with increased SNR of Z. This weak attenuation demonstrates that mvNPG can detect serial routing, yet it is not as suited for discriminating direct connectivity (i.e. X→ Y) from when there is relay via a secondary node (X → Z → Y).

**Fig. 9:**
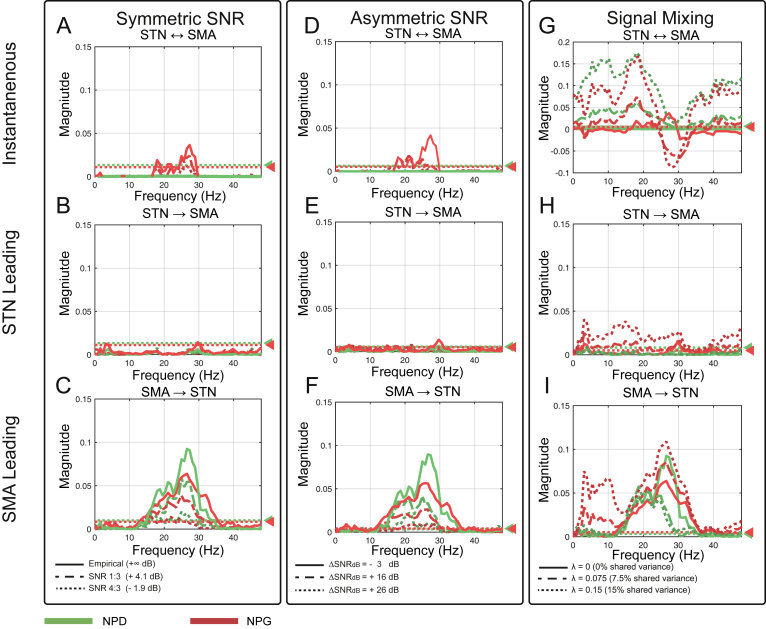
**Testing for the confounding effects of symmetric and asymmetric SNR, and instantaneous signal mixing upon estimation of directed functional connectivity in experimental data recorded in patients with Parkinson’s disease.** Empirical data is comprised of local field potentials recorded from the STN and a virtual electrode localized to the SMA, computed from whole-head magnetoencephalography. Signals were analysed for dFC using the instantaneous components (first row); STN → SMA (second row); and SMA → STN (third row) parts of the NPD (green) and NPG (red). Empirical data is indicated by bold line; low by the dotted (···); and high degrees by the dashed (---). **(A**–**C)** The effect of modulating the overall SNR of the signals equally. We used a range of narrowband (14–31 ​Hz) SNRs: 1:1 (+∞ dB; bold); 4:3 (+4.1 ​dB; ---); and 1:3 (−1.9 ​dB; ···). **(D**–**F)** The effect of modulating the SNR of the strongest signal (STN) only. We used a range of ΔSNR: −3 ​dB (bold); +16 ​dB (---); and +26 ​dB (···). **(G**–**I)** The effect of modulating the degree of instantaneous mixing between signals. We simulated a degree of signal mixing: *λ* ​= ​0 (0% shared variance; bold); *λ* ​= ​0.075 (7.5% shared variance; ---); and *λ* ​= ​0.15 (15% shared variance; ···).

We next looked at a feedforward network, where X propagates directly to Y, but is then relayed on to Z. Because some of the information passed X → Y is contained in Z, we expect conditioning to attenuate the directed connection. Again, we find that NPD(Z) behaves as expected, although the attenuation is weaker than in (A) when Z mediated the routing entirely. In this way the difference in values between the NPD and NPD(Z) yields a measure of how much information of X is fed forward from Y→ Z. Thus, decreasing SNR of the observation of Z decreases the attenuating effect of the conditioned NPD. mvNPG remains at a constant magnitude for all SNRs tested. This demonstrates that the multivariate estimator of Granger causality is not sensitive to feedforward configurations whereby the estimation of connectivity between X and Y is not influenced by activity at the terminal (receiving) node.

For the third test, we investigated the combination of recurrent loops in the network and incomplete signal observation-two features likely to occur in real recordings from neural systems. We find that with complete signal observation (i.e. SNR → ∞) the metrics behave similarly to the feedforward model. A notable difference is the increased NPD of X → Y compared to the feedforward case, as correlations are reinforced by signals resonating across the loop. NPD(Z) behaves in a similar way as before, showing attenuation of the conditioned estimate at low noise levels, but converging back to the unconditioned NPD as the reference signal is obscured by noise and estimation of its confounding influence is lost. The mvNPG estimate of the connection X → Y decreases by 0.1 as the observation noise of Z is reduced. This finding indicates that in the case of recurrent connectivity, mvNPG is sensitive to the quality of the signal recorded at the routing node. In the case of recurrent configurations, this finding shows that mvNPG can readily discriminate between direct X→ Y connectivity and cyclical routing via a secondary signal recorded at Z.

### 3.9 Example of estimation of directed functional connectivity in confounded empirical data: cortico-subthalamic connectivity

Using the example dataset described in section 2.6 we examine how changes in the overall SNR, differences in SNR between signals, and instantaneous signal mixing may act to confound the estimation of dFC in empirical data recorded from simultaneous MEG and LFP in patients with Parkinson’s disease. We first analyse the original empirical data and then subsequently introduce synthetic confounding effects as described in the methods section that outlined observation modelling. The results of this analysis are presented in Fig. 9.

We demonstrate in the original data that there is a clear asymmetry in coupling with both NPD and NPG indicating a clear dFC from SMA → STN. The zero-lag component (top row Fig. 9) of the NPD is negligible in the original data. In contrast, the instantaneous component of NPG shows above significance level connectivity at 20–30 ​Hz. The empirical SNR of the data was estimated using the method described in section 2.6.2. We use activity in the beta band (14–30 ​Hz) to define the signal and then compare with the noise floor. SNR estimates of the MEG virtual electrode and LFP were +1.9 ​dB (SNR_SMA_ ​≈ ​3:2) and +4.0 ​dB (SNR_STN_ ​≈ ​5:2) respectively. This yields an empirical ΔSNR of −2.1 ​dB with the LFP measured at the STN having the largest SNR.

In the first set of experiments we reduced the SNR of both signals equally (Fig. 9A–C). We added noise to the original signals to yield a range of SNRs: + ∞ dB, + 4.1 ​dB, and −1.9 ​dB. These analyses show that both NPG and NPD estimates of connectivity respond to a uniform reduction in SNR in a simple and predictable way by reducing their overall magnitude approaching zero as the signals become mostly noise.

Subsequently, the effect of changing the SNR of only one of the signals upon dFC estimates was also investigated (Fig. 9D–F). Signals were constructed to have a range of ΔSNR: 3 ​dB; +16 ​dB; and +26 ​dB. We reduced the SNR of the strongest signal only (STN; $SNR_{dB}^{STN}$), in an attempt to bias the directionality estimates in the reverse direction (i.e. increase the strength of STN → SMA). However, it was found for both NPD and NPG that this had a similar effect to reducing the SNR symmetrically (i.e. when SMA → STN is weakened). This result suggests that for this dataset, it may not be possible to induce a strong bias in the inferred dFC by making one signal weaker than the other (i.e. $SNR_{dB}^{SMA}$≫ $SNR_{dB}^{STN}$) as there is no anatomical STN→SMA feedback (a situation in contrast with simulations investigating asymmetric SNR in section 3.4.).

In the final column of Fig. 9 (panels G–I) the effect of signal mixing was measured. We simulated several degrees of signal mixing: *λ* ​= ​0 (0% shared variance); *λ* ​= ​0.075 (7.5% shared variance); and *λ* ​= ​0.15 (15% shared variance). Again, it was found that the instantaneous component of the NPD behaves as expected, increasing in magnitude with increased signal mixing. This is most apparent in the frequencies outside the main oscillatory bands of activity. When using the instantaneous part of NPG, we found that there was generally an increase, yet the frequencies around the main component (of the peak in the lagged connectivity) were negative and uninterpretable. Furthermore, we show that even moderate increases in the signal mixing (7.5%) corrupt the dFC estimation when using NPG. This is especially apparent at high mixing levels (15%), where a wide band reverse component (STN→SMA) arises, as well as large second peak in the SMA→STN at around 4–12 ​Hz. NPD estimates are much more stable in comparison and only show a reduction in the original peak with increased mixing, but no spurious peaks emerge outside of this range at any of mixing degrees tested.

## 4 Discussion

The results presented in this paper further support the NPD methodology as an accurate and robust method for the estimation of dFC in continuous neural data. We first provided a face validation of NPD for estimation of the directed interactions between MVAR processes. Secondly, we assessed the performance of the NPD measure in the presence of several confounding factors that are likely to arise in experimental recordings of neurophysiological networks, namely: volume conduction, common drive, low SNR, unequal SNRs between signals, and recurrent connectivity. Thirdly, we provided a direct comparison of NPD with a well-established estimate of dFC based on Granger causality – NPG. Finally, our results show that the additional information gained from using a conditioned, multivariate extension of the NPD method allows for some of the confounding influences of common drive, or non-trivial signal routing, to be mitigated. The degree to which this is achieved is dependent upon the extent to which the signal captures the neural activity at the recording site.

### 4.1 A summary of effects of signal confounds

#### 4.1.1 Effects of common drive

Common input to two parallel neural populations has long been known to be a confounding factor when estimating functional connectivity (Aertsen et al., 1989; Farmer et al., 1993; Horwitz, 2003). The limitations of finite sampling over the brain means that no FC measure is immune to this problem as there always remains the potential for an unmeasured common input to the recorded populations from which an FC estimate is made. Our simulations demonstrate this effect where both pairwise NPD and NPG estimates indicate spurious causality in the case of lagged common input. However, when using multivariate extensions of the two methods, in which the common drive signal is factored out, it is possible to avoid spurious estimation of connectivity between nodes sharing a common drive. This is shown to be true when using the multivariate NPG which accounts for the total covariance across the network. On the other hand, NPD in its simplest form is measured in a pairwise manner and cannot account for the action of a tertiary signal on the naïve estimate. However, we demonstrate that this issue can be remedied using the multivariate extension of NPD in which the influence of a common drive may be regressed out in order to eliminate spurious connectivity between the driven nodes. Whilst this is a solution when the common drive is observed, there still remains the potential confound of an unobserved common signal, to which NPD and NPG are equally susceptible. These issues can be addressed by model based estimators of effective connectivity such as dynamic causal modelling which allow for the inference of unobserved states in a causal network (Friston et al., 2013).

#### 4.1.2 Effects of A/symmetric signal-to-noise ratios

Functional connectivity estimates are subject to the limits of inference implied by the SNR of the available recordings. We demonstrate (Fig. 5A) that coherence, NPD, and NPG are degraded by poor SNR with similar logistic decays. However, NPG exhibits a greater susceptibility to degradation by noise than NPD. NPG magnitude are reduced at SNRs an order of magnitude higher than those that would elicit an equal reduction in NPD. Despite this, both measures show a remarkable resistance to even high levels of noise, with the range of SNRs at which both NPG and NPD provide statistically significant estimates of connectivity (i.e. having a magnitude exceeding the P ​= ​0.001 confidence interval) reaching as low as SNR ​= ​1:30 (equivalent to −15 dB). This would suggest that both are robust to the occurrence of false-negative errors as a result of poor SNR in neural recordings. These findings can also explain the common empirical finding of significant functional connectivity in the absence of obvious peaks in the power spectra.

A number of authors have noted that estimation of Granger causality is biased by the existence of unequal SNRs (Bastos and Schoffelen, 2016; Haufe et al., 2013; Nolte et al., 2008). Our simulations reiterate this fact and demonstrate that NPG is biased to estimate the driving node as the strongest signal (section 3.4; Fig. 5B). This is an important problem as all neurophysiological signals comprise some unknown mixture of the signal of interest and background noise on a source by source basis. As a result, it can rarely be assumed that the SNRs of two signals are balanced. We find that when investigating the simultaneous effects of unequal SNR and instantaneous mixing, that NPG is most easily corrupted by the asymmetry in the signals, whereas NPD is most sensitive to mixing.

This is particularly important when looking at directed connectivity between signals recorded from two different modalities (e.g. MEG and LFP) where the estimate will be biased in favour of the higher gain recording (to lead). For instance, in our data we show a difference in empirical SNRs of 4.5 ​dB between the LFP and virtual channel signals. This has led some authors to suggest the usage of time-reversed data as surrogate comparison for dFC methods (Haufe et al., 2013) because if a true causal effect is present then time reversal should flip the sign of the directionality. Future validations should explore whether this approach can reduce the susceptibility of the NPD method to so called weak” data asymmetries. However, the simulations here demonstrate that estimates made using NPD are far less subject to this confound than NPG. NPD is still affected by decreased SNR (both asymmetric and symmetric) but shows no bias, as directional estimates decrease uniformly as the SNR goes down. This finding leads us to suggest that in future studies of dFC in multimodal data or in other cases where the signals are likely to be of differing SNR, the NPD method provides a more robust and readily interpretable result over Granger based approaches.

#### 4.1.3 Effects of simulated volume conduction through signal mixing

The extent to which signals recorded from the brain are subject to the influence of volume conduction is generally more severe with decreasing distance between the recording electrodes. Experiments have demonstrated that LFPs measured from electrodes separated by a distance of 5 ​cm will typically show R^2^ values indicating approximately 50% shared variance (Nunez et al., 1997) and so analyses of directed functional connectivity are likely to be significantly affected by instantaneous mixing at distances much closer than this (e.g. recordings made from neighbouring contacts of the same intracranial electrodes). Instead, some authors have shown that functional connectivity analyses are better suited to source localized signals due to the reduced extent of signal leakage (Schoffelen and Gross, 2009). This is likely to hold true for the application of NPD analysis to whole brain recordings. It is difficult to find a limit for when zero-lag effects will corrupt a method such as NPG as this ultimately depends on the nature of the lagged connectivity present in the data. In our simulations, we show that the bias on NPG induced by mixing is dependent upon the original SNR of the signals as a result of confounding by the mechanism of SNR asymmetry discussed in the previous section.

In addition to the benefit of being less susceptible to corruption by volume conduction, NPD provides explicit frequency resolved estimation of the zero-lag component of coherence, making it possible to estimate the extent to which coupling is influenced by instantaneous effects. This characteristic affords NPD an advantage over corrected methods of FC such as imaginary coherence or the phase locking index (Nolte et al., 2004; Lachaux et al., 1999; Vinck et al., 2011) which are set up to ignore zero-phase coherence. In this respect it is important to note that zero-phase coherence can reflect synchronous physiological coupling (Roelfsema et al., 1997). We also note that volume conduction is manifest not only through the mixing of known sources of interest, but also hidden sources (Bastos and Schoffelen, 2016). This introduces a confound like that of limited signal observation (see below) in which the influence of a node can only be estimated if it is directly observable.

#### 4.1.4 Effects of data length on estimation accuracy

Estimation of connectivity in empirical data is limited by the availability of recorded data due either to experimental design or practical limitations of storage and acquisition. In Fig. 6 we presented a set of tests to determine the sensitivity of the two methods to data length. Overall, we find that NPG is the most robust of the two and can make accurate recovery at trial lengths two orders (to the power of 2) shorter than NPD. As expected, this exact effect is dependent upon the complexity of the model to be evaluated. In the case where more dense networks are to be estimated, the required amount of data is larger than for simpler models with one or two connections. These limitations are likely due to the variance of the spectral estimators from which the two metrics are decomposed, with coherence known to be sensitive to having a larger number of samples to restrict estimates.

It is well known that insufficient sampling will hinder parametric estimators of Granger causality (Seth et al., 2015). Simulations here suggest that with a good number of trial repetitions (>35) at a sampling rate of 200 ​Hz, trial lengths from 0.5 to 1 ​s are required for accurate estimation with NPD. For NPG, this requirement is reduced to a minimum of 0.2 ​s, although these guidelines are likely to depend upon the SNR, as well as frequency band of the interaction of interest in the analysed data.

#### 4.1.5 Effects of limited signal observation

The argument that conditioned metrics of dFC such as conditioned NPD provide an increased ability to infer the causal structure of real-world neural networks hinges upon the assumption that a recorded signal truly captures the complete dynamics of the underlying population through which the signal is routed. In section 3.8 we provide an analysis of how the incomplete observation of signals acts to confound the estimates of dFC under several hypotheses of signal propagation: A) serial; B) feedforward; and C) recurrent connectivity (Fig. 8). In the case of the simplest architecture - serial propagation, the metrics behave as expected – the more poorly the signal used to perform the conditioning captures the underlying dynamics, the less the conditioning can inform accurate estimation of directed connectivity. In the case of complete signal capture, the conditioning procedure (NPD(Z)) completely attenuates the directed connection (X → Y), as there is no possibility that any of the information contained in Y concerning node X is exclusive of Z. Therefore, if the signal recorded at Z completely captures the dynamics of Y then there is the potential to attenuate the X → Y connection entirely using a conditioning on Z. In the case of feedforward propagation, conditioning will also act to attenuate the estimate, but unlike serial processing (where the reference node Z provides an intermediate node in the chain of propagation between X and Y) the attenuation can never be complete as the variance introduced at Z is not shared with X or Y. In case C we show that the re-entrant connection acts to increase the overall coherence due to cyclical passage of information in the circuit. Furthermore, conditioning acts to bring the NPD estimate closer to that of the feedforward model (where the re-entrant connection is missing). In the case of recurrent connectivity, the multivariate NPG also acts to discount the reconnection via Z. When Z is completely captured (SNR is high) then the NPG gives an estimated connectivity equivalent again to the feedforward model.

These observations make it clear that if conditioning removes the inferred connectivity in its entirety then the conditioned node must be in a relay like position (i.e. X → Z → Y). For instance, this was found to be the case in West et al. (2018) where conditioning of NPD between the striatum and subthalamic nucleus upon the external segment of the globus pallidus removed connectivity almost entirely, leading to the conclusion that information propagated serially in the network, a finding in-line with known anatomical details of the indirect pathway of the basal-ganglia. However, the findings described in the present paper regarding the combination of circuit organization and SNR of conditioned signals introduce ambiguity when interpreting the results of conditioned or multivariate estimates of directed connectivity in empirical data. For instance, incomplete attenuation of conditioning may arise either from poor SNR of the reference signal in a serial network or may indicate that the conditioned signal is placed in either a feedforward or recurrent configuration. In this case it is necessary to combine evidence from multiple conditioning steps (e.g. also conditioning X → Z on Y) in order to determine the exact signal routing. Previous work has argued that additive noise only impairs estimation rather than distorts temporal structure of the signals (Baccalá and Sameshima, 2006), here we show that this disruption is dependent upon the exact routing of the signals. Specifically, in networks containing a high degree of reciprocity, partialized estimated of coherence (both directed and undirected) are likely to be confounded. This finding could be used in principle to further specify the role of a conditioned node by determining its effect upon directed connectivity in response to additive noise.

### 4.2 Extensions and final conclusions

We have presented a validation of NPD, a novel tool for the assessment of dFC, in continuous neural recordings such as that measured in methods commonly used for human neuroimaging. We argue that in the face of common practical issues arising from the physical limitations of many experimental recording methods, as well as from the complex biology of the systems that they aim to explore, NPD and its conditioned extension provide a useful method that builds upon the founding principles of the more established Granger causality. The NPD measure (conditioned and unconditioned) has been recently demonstrated to provide insights into the patterns of propagating neural activity in animal electrophysiology in the basal-ganglia (West et al., 2018) and hippocampus (Halliday et al., 2016); as well as in human motor networks (Halliday, 2015; Spedden et al., 2019). and is likely to have wide application across other domains of clinical and experimental neuroscience. The finding that NPD is robust to the confounding effects of SNR asymmetry means that it may be readily applied to multi-modal neural recordings without some of the concerns that may arise with Granger-based methods.

The validation provided here is not extensive: there is a wide range of other existing dFC metrics to which we have not made comparison, and so it is possible that other metrics may perform better than NPG (for an extensive comparison of many metrics, not including NPD, see Wang et al., 2014). Granger causality-based methods have become a staple of the dFC toolbox and form the statistical foundation for several methods developed since including the directed transfer function (Kaminski and Blinowska, 1991) and partial directed coherence (Baccalá and Sameshima, 2001). An adaptation of the directed transfer function aimed at improving estimation of directed connectivity (i.e. *X* → *Y*) introduced by Korzeniewska et al., (2003) may perform better at recovering known patterns of connectivity in the face of common drive than the metrics presented here. Furthermore, the role of time reversal procedures (Haufe et al., 2013) in alleviating some of these shortcomings in the metrics should be the subject of future study. This is likely to be important when investigating more complex networks or high dimensional data such as that measured with magneto- or electroencephalographic recordings. However, NPD shows broadly equivalent results to the Granger based measure but exhibits more robust performance in the recovery of complex network topologies in highly confounded data. The full extent to which this is true either in networks of a greater size or density will need to be tested.

We conclude that the NPD measure of directed functional connectivity is inexpensive to compute, makes limited assumptions of the properties of the data, is flexible to the form of the original spectral estimate and is conceptually simple to formulate. It eschews the computationally expensive estimation of model parameters required for parametric estimates of Granger causality or directed transfer function and doesn’t require iterative binning procedures such as that use in information-based metrics like transfer entropy. Overall, NPD provides a simple and compact statistical description of directed dependencies between signals and is readily interpretable, providing the basis for testable hypotheses of causation in real neural systems.

## Funding

S.F.F. receives funding from UCLH BRC. Engineering Research Council UK (awards EPSRC EP/F500351/1 to T.O.W). 10.13039/501100000265Medical Research Council UK (award MR/R020418/1 to support T.O.W) The 10.13039/100010269Wellcome Trust (ref: 204829) through the Centre for Future Health (CFH) at the University of York to D.H. The Wellcome Centre for Human Neuroimaging is supported by core funding from the 10.13039/100010269Wellcome 203147/Z/16/Z. UK MEG community is supported by the MRC UKMEG Partnership grant MR/K005464/1.

## CRediT authorship contribution statement

**Timothy O. West:** Conceptualization, Methodology, Software, Writing - original draft, Writing - review & editing. **David M. Halliday:** Methodology, Software, Writing - review & editing. **Steven L. Bressler:** Conceptualization, Supervision, Writing - review & editing. **Simon F. Farmer:** Conceptualization, Supervision, Writing - original draft, Writing - review & editing. **Vladimir Litvak:** Conceptualization, Resources, Supervision, Writing - original draft, Writing - review & editing.

## Declaration of competing interest

The authors have no competing interests to declare.

## Figures and Tables

**Table 1.1:** Table of MVAR Coefficients


Simulating Common Drive, Signal-to-noise, instantaneous mixing (Fig. 1, Fig. 2, Fig. 4, Fig. 5C)	
*Transfer Matrix*	*Noise Covariance Matrix*
$A_{1}=\left[\begin{array}{ccc} 0.5 & 0 & 0\\ 0 & 0.5 & 0\\ 0 & 0 & 0.5 \end{array}\right]$ $A_{2}=\left[\begin{array}{ccc} -0.5 & 0 & 0\\ 0.5 & -0.5 & 0\\ 0 & 0 & -0.5 \end{array}\right]$ $A_{3}=\left[\begin{array}{ccc} 0.5 & 0 & 0\\ 0 & 0.5 & 0\\ 0.5 & 0 & 0.5 \end{array}\right]$	$\varepsilon =\left[\begin{array}{ccc} 0.3 & 0 & 0\\ 0 & 0.3 & 0\\ 0 & 0 & 0.3 \end{array}\right]$
**Simulating Asymmetric Signal-to-noise (**Fig. 3, Fig. 5B**)**	
*Transfer Matrix*	*Noise Covariance Matrix*
$A_{1}=\left[\begin{array}{cc} 0.5 & 0\\ 0 & 0.5 \end{array}\right]$ $A_{2}=\left[\begin{array}{cc} -0.5 & 0.35\\ 0.35 & -0.5 \end{array}\right]$ $A_{3}=\left[\begin{array}{cc} 0.5 & 0\\ 0 & 0.5 \end{array}\right]$	$\varepsilon =\left[\begin{array}{cc} 0.3 & 0\\ 0 & 0.3 \end{array}\right]$
**Simulating Incomplete Signals for Conditioning: Serial (**Fig. 8A, D**)**	
*Transfer Matrix*	*Noise Covariance Matrix*
$A_{1}=\left[\begin{array}{ccc} 0.5 & 0 & 0\\ 0 & 0.5 & 0\\ 0 & 0 & 0.5 \end{array}\right]$ $A_{2}=\left[\begin{array}{ccc} -0.5 & 0 & 0\\ 0 & -0.5 & 0\\ 0 & 0 & -0.5 \end{array}\right]$ $A_{3}=\left[\begin{array}{ccc} 0 & 0 & 0\\ 0.3 & 0 & 0\\ 0 & 0.3 & 0 \end{array}\right]$	$\varepsilon =\left[\begin{array}{ccc} 0.3 & 0 & 0\\ 0 & 0.3 & 0\\ 0 & 0 & 0.3 \end{array}\right]$
**Simulating Incomplete Signals for Conditioning: Feedforward (**Fig. 8B, E**)**	
*Transfer Matrix*	*Noise Covariance Matrix*
$A_{1}=\left[\begin{array}{ccc} 0.5 & 0 & 0\\ 0 & 0.5 & 0\\ 0 & 0 & 0.5 \end{array}\right]$ $A_{2}=\left[\begin{array}{ccc} -0.5 & 0 & 0\\ 0 & -0.5 & 0\\ 0 & 0 & -0.5 \end{array}\right]$ $A_{3}=\left[\begin{array}{ccc} 0.5 & 0 & 0\\ 0 & 0.5 & 0\\ 0.3 & 0 & 0.5 \end{array}\right]$	$\varepsilon =\left[\begin{array}{ccc} 0.3 & 0 & 0\\ 0 & 0.3 & 0\\ 0 & 0 & 0.3 \end{array}\right]$
**Simulating Incomplete Signals for Conditioning: Recurrent (**Fig. 6C, F)	
*Transfer Matrix*	*Noise Covariance Matrix*
$A_{1}=\left[\begin{array}{ccc} 0.5 & 0 & 0\\ 0 & 0.5 & 0\\ 0 & 0 & 0.5 \end{array}\right]$ $A_{2}=\left[\begin{array}{ccc} -0.5 & 0 & 0\\ 0 & -0.5 & 0\\ 0 & 0 & -0.5 \end{array}\right]$ $A_{3}=\left[\begin{array}{ccc} 0 & 0.3 & 0\\ 0 & 0 & 0.3\\ 0.3 & 0 & 0 \end{array}\right]$	$\varepsilon =\left[\begin{array}{ccc} 0.3 & 0 & 0\\ 0 & 0.3 & 0\\ 0 & 0 & 0.3 \end{array}\right]$

## Appendices

### List of External Toolboxes Used in Analysis, Statistics, and Plotting

1. BSMART toolbox- Hualou Liang, Steven Bressler, Mingzhou Ding (Cui et al., 2008): http://www.brain-smart.org/
2. Fieldtrip toolbox- Donders Insitute, Radboud University (Oostenveld et al., 2011a): www.fieldtriptoolbox.org/
3. Linspecer - David Kun: https://github.com/davidkun/linspecer
4. Neurospec 2.11 toolbox- David Halliday, University of York: http://www.neurospec.org/
5. SPM 12 toolbox- UCL, Wellcome Centre for Human Neuroscience: https://www.fil.ion.ucl.ac.uk/spm/

### Permutation Testing for Significance Thresholds

Estimates of interactions between signals can give rise to non-zero values in the absence of any interaction. To check whether an observed value of an estimator is statistically significant, its value can be compared against a distribution of the same index estimated from surrogate data (Theiler et al., 1992). This surrogate data must preserve the statistics of the individual signals whilst removing the property to be tested, namely the interactions between them. Once a null distribution is obtained, (rank-order) significance thresholds can be calculated from the corresponding percentiles of the surrogate distribution (e.g. a P ​= ​0.01 threshold can be estimated from the 99th percentile).

Many possibilities exist for the generation of surrogates and methods range from the very simple permutation of the time series samples, up to increasingly complex surrogates that aim to maintain nonlinear features of the individual signals (for reviews see: Lancaster et al., 2018; Pereda et al., 2005). All methods will fall short at capturing all confounding properties of the individual generators of the signals including the phase randomization method (Zalesky and Breakspear, 2015).

In this work we use a phase randomization approach. We maintain the original FFTs of the signals but randomize the phase of the individual Fourier components. To do this we generate a vector of random phases Ψ uniformly sampled on the interval [0 2π] for N/2 samples and multiply the first half of the FFT by exp (iΨ). The remainder of the FFT is then the horizontally flipped complex conjugate of the first half. This method ensures that the overall expected value of the magnitude of the averaged spectral estimate is equal to zero. See Lancaster et al. (2018) for a discussion of this method and Breakspear and Terry (2002) for application to assessing interdependence of neurophysiological signals.
